# Efficacy of Dexamethasone Diluted Saline Irrigant on Postoperative Sequelae in Patients Undergoing Lower Third Molar Surgery: A Prospective Clinical Study

**DOI:** 10.7759/cureus.45436

**Published:** 2023-09-18

**Authors:** Soorumsetty Ruthvik, Murugesan Krishnan, Melvin George, Santhosh P Kumar, Saravanan Lakshmanan

**Affiliations:** 1 Oral and Maxillofacial Surgery, Saveetha Dental College and Hospitals, Saveetha Institute of Medical and Technical Sciences, Saveetha University, Chennai, IND

**Keywords:** third molar surgery, irrigation, plain saline, dexamethasone sodium phosphate, postoperative sequelae

## Abstract

Background

Third molar impaction surgery is one of the most common yet challenging procedures done as a part of minor oral surgery. Years of research and improvisation of techniques have been done, yet there are still a lot of postoperative sequelae after surgical removal of the impacted tooth. In our study, we have compared the efficacy of dexamethasone diluted saline solution over plain saline solution used as an irrigant in the reduction of postoperative sequelae for lower third molar surgery.

Aim

The aim of the study was to evaluate the efficacy of dexamethasone diluted saline solution over plain saline solution in the reduction of postoperative sequelae for lower third molar surgery.

Materials and methods

The research was conducted at Saveetha Dental College and Hospital in the Department of Oral and Maxillofacial Surgery. The study consisted of 48 individuals, 24 of whom had dexamethasone saline as an irrigant (8 mg of dexamethasone was diluted in 100 ml of plain saline) (Group 1), and 24 in whom plain saline was used as an irrigant (Group 2) in the lower third molar surgery. Patients were evaluated postoperatively for pain and swelling. The postoperative swelling was measured on postoperative day two and day seven. Postoperative pain was measured on day two, day four, and day seven after surgery using a visual analog scale. Data were analyzed using SPSS (IBM Corp., Armonk, NY) with P-values less than 0.05 considered statistically significant. The statistical test used to compare the outcomes between the two groups was the independent samples t-test.

Results

It was found that study participants in the dexamethasone saline irrigation group reported statistically significantly lesser pain than participants receiving plain saline irrigation on day two (P = 0.001), day four (P = 0.001), and day seven (P = 0.001), respectively. Also, there was a reduction in swelling among participants in the dexamethasone saline irrigation group when compared to the normal saline irrigation group, which was statistically significant (P = 0.001) on day two, while the postoperative swelling was not statistically significant on day seven (P = 0.08) between the two study groups.

Conclusion

Based on the results obtained, it can be concluded that dexamethasone saline solution (8 mg/100 mL) was more effective as an irrigant in reducing the postoperative sequelae than regular saline solution in the lower third molar surgery.

## Introduction

Historical evidence shows that treatment for decayed teeth was done from 10,000-4500 BC. It is shocking to know that barbers in Europe in the late 1700s developed most of the instruments like forceps and elevators for the removal of teeth [1]. Techniques to remove the impacted third molars have gained popularity only at the end of the 18th century. Initially, third molar impaction surgeries were performed with the help of chisel and mallet, which caused a lot of traumas to the adjacent soft and hard tissues and led to a lot of postoperative sequelae. It was considered one of the most painful procedures by the early British. Over a period of time, a lot of new developments happened in the removal of impacted third molar surgery, which has reduced most of the postoperative sequela [2].

Surgical removal of the impacted third molar is the most common yet one of the most challenging procedures in minor oral surgery performed worldwide. Even though it is the most common procedure performed, most concepts regarding third molar surgery have been unclear and have raised many opinions over the past few decades [3]. A lot of literature has been already published on the concepts of surgical removal of impacted third molar, but still, it is very surprising to know that a lot of concepts are still very unclear [2-4]. Of all the literature available, most of it is concentrated on the management or prevention of postoperative complications after third molar surgery, rather than techniques and methods for removal of impacted third molar. Contemporary oral and maxillofacial surgery aims at reducing the postoperative effects through a wide range of modalities. The modalities that have been currently in use are analgesics. Initially, analgesics have been given postoperatively to reduce postoperative pain, whereas recently surgeons have started to give preemptive analgesics as a safety measure to prevent immediate discomfort after the third molar surgery [5].

Flap designs that are less invasive gave better results with minimal postoperative swelling and trismus. According to a comparative study between different flap designs for third molar surgery, the author claimed that there is no significant difference in the relation between the flap design and postoperative sequela [6]. Other modalities in the reduction of postoperative sequelae include corticosteroids, sutures, and additional therapies like ozone therapy, cryotherapy, platelet-rich plasma and fibrin, the use of lasers, and piezoelectric surgery. While many techniques have been used for the reduction of postoperative sequela, basic techniques like the use of proper copious saline to irrigate while tooth splitting and bone guttering can reduce most of the complications like alveolar osteitis, and also reduce postoperative swelling, trismus, and pain [7].

Steroid medication like dexamethasone is one such potent drug, which is long known for its anti-inflammatory properties, reducing postoperative swelling. Dexamethasone has been shown to reduce swelling, pain, and trismus [8]. There are studies that involved the administration of dexamethasone intramuscularly and submucosally. The irrational use of dexamethasone can lead to adrenal insufficiency. Since the action of the drug is required in a local site, systemic administration may not be required. Side effects with the submucosal route have fewer complications compared to the intramuscular route [9]. As an alternative, we have used dexamethasone 8 mg with saline as an irrigant to evaluate its potential in reducing postoperative sequelae for lower third molar surgery.

The aim of the study was to evaluate the efficacy of dexamethasone diluted saline solution over plain saline solution in the reduction of postoperative sequelae for lower third molar surgery.

## Materials and methods

Study design

This prospective comparative study was conducted in the Department of Oral and Maxillofacial Surgery, Saveetha Dental College and Hospital. The time frame of the study was from October 2022 to April 2023. As per G*Power calculation, the study required a total of 44 patients with 95% power. To compensate for the loss to follow-up, an additional four samples were included in the study, making the total sample size of 48. A prior proforma with informed consent was obtained from all the patients included in the study. A total of two groups with 24 samples in each group were allocated using a simple random sampling technique. Allocation was done using an opaque envelope. Surgical removal of impacted teeth was done under local anesthesia by the same surgeon and assessments were also done by the same principal investigator in all the groups. Facial measurements were taken preoperatively in all the patients.

Inclusion criteria

All the patients irrespective of sex, with ages ranging from 20 to 40 years, who wanted prophylactic removal of impacted mandibular third molars were included in the study.

Exclusion criteria

Patients with existing systemic illnesses like uncontrolled diabetes and hypertension, and grossly decayed third molars with complaints of acute pain were not included in the study.

Surgical procedure

Under aseptic sterile conditions, standard scrubbing was done and the patients' intra-oral site was prepared. Local anesthesia with 1:80,000 dilution with adrenaline was given as inferior alveolar nerve block on the side of tooth removal, modified wards incision was used in all the patients, and full thickness mucoperiosteal flap elevated and bone guttering was done according to Moore and Gillbe collar technique. In Group 1 patients, 8 mg of dexamethasone was diluted in 100 ml saline and used as an irrigant, and in Group 2 patients, 100 ml of plain saline at room temperature was used as an irrigant during lower third molar surgery. Double blinding was followed in this study, in which both operator and patient were not aware of the allocated irrigant. The tooth was removed, hemostasis was obtained, closure was done using 3-0 silk, and immediate postoperative medications were given as per standard protocol.

Follow-up

**Figure 1:**
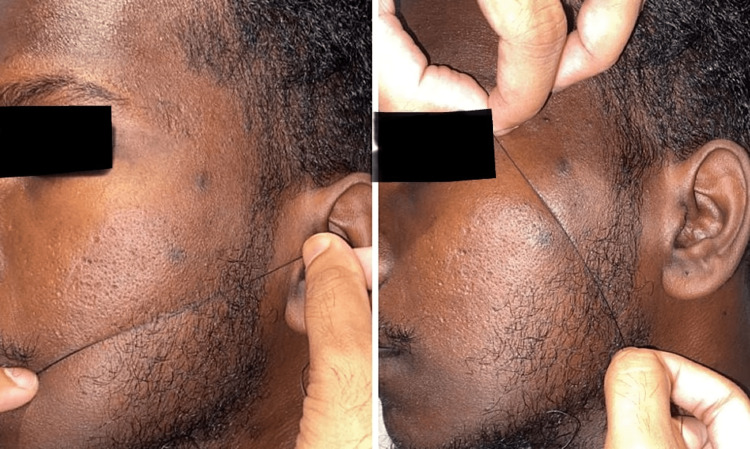
Facial measurement of swelling using four stable points over the face

Two parameters that were assessed in the postoperative follow-up period were postoperative pain and postoperative swelling and they were compared between groups. Postoperative pain was assessed postoperatively using a 10-point visual analog scale on day two, day four, and day seven postoperatively. Facial measurement of swelling was done using four-point swelling measurement, which includes measurement from the corner of the mouth to the tragus and corner of the eye to soft tissue gonion, as shown in Figure 1 [10]. Facial swelling was measured preoperatively and on postoperative day two and postoperative day seven.

Statistical analysis

The data were collected and analyzed with means and standard deviations with a 95% confidence interval. SPSS software version 23.0 (IBM Corp., Armonk, NY) was used for statistical analysis, and an unpaired t-test was used to assess the continuous variables between the two groups. The normality between the two groups was checked using the Shapiro-Wilk test. Results were considered statistically significant if the p-value was less than 0.05.

## Results

Our study population consisted of 26 males and 22 females with a mean age of 26 ± 5.3 years. The present study includes two groups: Group 1 (N = 24), in which 8 mg dexamethasone diluted in 100 ml saline was used as an irrigant solution; Group 2 (N = 24), in which plain saline was used as an irrigant solution in the lower third molar surgery. Postoperative pain and swelling were measured in all the patients included in the study at regular follow-up intervals.

Pain measurement

Pain measurement was done with the help of a 10-point visual analog scale, recorded at intervals of postoperative day two, day four, and day seven. The values obtained are depicted in Table 1.

**Table 1:** Comparison of pain scores among the two study groups Group 1 - Dexamethasone saline group; Group 2 - Plain saline group. ** Statistically significant; independent sample t-test.

Visual analog scale	Groups	N	Mean	SD	P-value
Day 2	Group 1	24	4.13	0.79	0.001**
	Group 2	24	5.1	1.17	
Day 4	Group 1	24	2.46	1.02	0.001**
	Group 2	24	3.42	0.95	
Day 7	Group 1	24	1.17	0.86	0.001**
	Group 2	24	2.17	0.702	

Based on the results, it was found that study participants in the dexamethasone saline irrigation group reported statistically significantly lesser pain than participants receiving plain saline irrigation on day two (P = 0.001), day four (P = 0.001), and day seven (P = 0.001), respectively.

Measurement of swelling

Facial measurements were taken preoperatively and then postoperatively on day two and day seven using a four-point measurement scale. The mean of two measurements was considered for assessment. The values obtained are depicted in Table 2.

**Table 2:** Comparison of swelling values among the two study groups Group 1 - Dexamethasone saline group; Group 2 - Plain saline group. ** Statistically significant; NS - not significant; independent sample t-test.

Swelling	Groups	N	Mean	SD	P-value
Preoperative	Group 1	24	9.75	0.314	0.23
	Group 2	24	9.63	0.373	NS
Day 2	Group 1	24	10.12	0.305	0.001**
	Group 2	24	10.9	0.309	
Day 7	Group 1	24	9.82	0.27	0.08
	Group 2	24	9.98	0.33	NS

Based on the results obtained, it was found that there was a reduction in swelling among participants in the dexamethasone saline irrigation group when compared to the normal saline irrigation group, which was statistically significant (P = 0.001) on day two, while the postoperative swelling was not statistically significant on day seven (P = 0.08) between the two study groups.

## Discussion

Even though surgical removal of impacted lower third molars is the most common procedure done as a part of minor oral surgery, there is a lot more to master in this procedure so that most of the postoperative sequelae like pain, swelling, and trismus can be reduced. Postoperative pain and swelling are two major reasons for patients hesitating to get the third molar surgically removed. Generally, postoperative pain and swelling subside in three to four days of the time period, but it is this time period when patients have maximum complaints and anxiousness [11].

A lot of studies have been done to reduce postoperative discomfort to the patient mainly in those initial three to four days of time period [3,5]. The use of analgesics postoperatively has much relief to the patient, but the pain relief was only transient, and at any particular point of time in the day, the patient experienced pain before taking the next dose of analgesic medication. To reduce postoperative swelling, postoperative corticosteroids have been administered to the patient [11]. Mostly the route of administration would be intravenous or intramuscular. There have been studies where steroids like dexamethasone have been administered intramucosally at the site of surgery preoperatively or in the immediate postoperative period and these studies demonstrated reduced postoperative swelling, pain, and trismus and results obtained were statistically significant [12-14].

Some studies have also proved that dexamethasone has an inhibitory effect on prostaglandins, hence reducing postoperative pain [13-15]. In a clinical trial, it was proved that dexamethasone has more beneficial effects than other corticosteroid drugs like methylprednisolone in pain reduction and swelling control for lower third molar surgery [16]. In all the existing studies, dexamethasone is given either intravenously or locally using intramuscular or intramucosal routes [16-19]. Our study evaluated the beneficial effects of dexamethasone if diluted in saline and used as an irrigant in lower third molar surgery, which is non-invasive and comfortable for the patients.

In our study, we obtained a statistically significant reduction in pain scores postoperatively in the dexamethasone saline irrigant group when compared with the plain saline irrigant group. The pain relief was very instant, and patients reported very low visual analog scale scores on immediate postoperative day two in the dexamethasone saline group while in the plain saline group, pain reduction was only evident in the seventh postoperative day. Postoperative swelling was very low in the dexamethasone saline irrigant group on the second postoperative day when compared with the plain saline irrigant group and the values were also statistically significant. No statistically significant difference between the groups in swelling values was noticed on the postoperative seventh day.

Limitations of the study

Although this study proved that there is a significant reduction in pain and swelling when dexamethasone in saline is used as an irrigant, the only drawback of this study was its limited sample size. Further studies with larger sample sizes can be done so that the efficacy of dexamethasone in saline as an irrigant can be properly analyzed, and the results can be generalized.

## Conclusions

From the results obtained, it is evident that usage of 8 mg dexamethasone diluted in 100 ml of saline as an irrigant can very effectively reduce postoperative swelling and pain when compared to plain saline irrigant in patients undergoing third molar surgery. Since dexamethasone is a very economical and easily available drug, it can be used as an irrigant in regular practice for third molar surgery.
